# Life expectancy and healthy life expectancy of Korean registered disabled by disability type in 2014–2018: Korea National Rehabilitation Center database

**DOI:** 10.1186/s12889-023-16682-9

**Published:** 2023-09-08

**Authors:** Hyuna Jang, Kyung-Hwa Choi, Jung-Ae Kim, Yong-jun Choi

**Affiliations:** 1 Department of Statistics, Sookmyung Women’s University, Seoul, Korea; 2 Department of Preventive Medicine, Dankook University College of Medicine, 119 Dandaero, Dongnam-Gu, Cheonan, Chungnam 31116 Republic of Korea; 3 Department of Nursing, Kyungbok University, Namyangju, Korea; 4 Department of Social and Preventive Medicine, Hallym University College of Medicine, Chuncheon, Gangwon Korea; 5 Institute of Health Services, Hallym University College of Medicine, Chuncheon, Gangwon Korea

**Keywords:** Life tables, Life expectancy, Healthy life expectancy, Disabled persons

## Abstract

**Background:**

Conducting a distinct comparison between the life expectancy (LE) and healthy life expectancy (HALE) of people with disabilities (PWDs) and the general population is necessary due to the various environmental and health conditions they encounter. Therefore, this study aimed to develop the life table for PWDs and calculate those of LE and HALE based on sex, severity, and disability types among the registered Korean PWDs.

**Methods:**

We used aggregated data of registered PWDs from the Korea National Rehabilitation Center database between 2014 and 2018. Overall, 345,595 deaths were included among 12,627,428 registered PWDs. First, we calculated the LE for total PWDs and non-disabled people using a standard life table, extending the old age mortality among nine models. Subsequently, we calculated the LE for each type of disability using the relationship between the mortality of total PWDs and those of each type of disability. Finally, HALE was calculated using the Sullivan method for three types as follows: disability-free and perceived health (PH) using the National Survey, and hospitalized for  ≥ 7 days using the Korea National Health Insurance System (NHIS) database.

**Results:**

The calculated LE/HALE–NHIS (years) at registration in males and females were 81.32/73.32 and 87.38/75.58, 68.54/58.98 and 71.43/59.24, 73.87/65.43 and 78.25/67.51, and 61.53/50.48 and 62.41/49.72 years among non-disabled, total PWDs, mild disabled, and severe disabled, respectively. LE/HALE-NHIS was lowest and highest in respiratory dysfunction and hearing disabilities, respectively.

**Conclusions:**

Males with disabilities had shorter LE and HALE at registration than females, except for those with severe disabilities, and there were variabilities in the LE based on the disability types.

**Supplementary Information:**

The online version contains supplementary material available at 10.1186/s12889-023-16682-9.

## Background

People with disabilities (PWDs) have limitations in one or more major life activities. They differ from non-disabled people (ND) in socioeconomic and demographic characteristics depending on the disability [1]. PWDs have a greater mortality risk than the general population [2] and a higher prevalence of chronic diseases [1], with usually more than one chronic condition simultaneously [3]. Furthermore, they have limited access to preventive and curative health services and discrimination in healthcare utilization [4]. Therefore, investigating the health gap between PWDs and NDs due to these diverse environmental and health conditions is crucial.

Given the growing attention towards PWDs in Korea, the Ministry of Health and Social Affairs launched a pilot program that focused on the registration of disabilities and severity examination system in 1987, which subsequently expanded nationwide in 1988 [5]. The law defined the disability type, diagnostic institutions, specialists, and diagnosis periods in the Enforcement Decree of the Act On the Welfare of PWDs [6]. According to official statistics, 2,622,950 PWDs were registered in 2020, accounting for approximately 5% of the total population [7]. Additionally, the disability enrollment rate is consistently approximately 95% of the total PWDs [8]. While the World Health Organization defines the PWDs as those with impairments, activity limitations, and participation restrictions due to the negative interaction between their health condition and contextual factors (environmental and personal factors) [9], the Korean system focuses on administrative convenience; therefore, the criteria for judgment are based on medical conditions. Furthermore, the registration barriers and severity of disability in Korea are higher than those in other countries, and the scope of welfare benefits is limited [10]. Therefore, studies on PWDs in Korea should compare the characteristics of each disability type among registered PWDs rather than focusing on a single disease.

Life expectancy (LE) and healthy life expectancy (HALE) are global key metrics for monitoring health and indicating the health gap among countries [10]. These indicators can assess the mortality patterns [11] and monitor changes in health quality at the national long-term health level [12]. Therefore, LE and HALE can be used as key indicators, reflecting the health gap between PWDs and ND.

In a few countries, LE and HALE studies among PWDs and NDs have been conducting [10]. A few of those studies compared disability type and severity [13–18]: In the United States, LE in cerebral palsy [13], LE and HALE of patients with Alzheimer's disease [14], and LE of 1-year survivors of traumatic brain injury [15] were reported; In Japan, Chiu CT et al. [16] examined the influence of strokes on a disabled or disability-free life for elderly using life expectancy; In Korea, Bahk J et al. [17] described the proportional mortality ratio and calculated life expectancy according to disability type; In England, Jagger C et al. [18] investigated how various HALE have changed between 1991 and 2011; In Brazil, Belon AP et al. [19] examined HALE among elderly using a self-reported functional disability. Most of those studies follow the general life table that does not reflect the disability type or cover the total PWDs.

Therefore, this study aimed to compile a standard life table for registered PWDs and calculate the LE and HALE based on disability types, severity, and sex using the standard model.

## Methods

### Study populations

We analyzed the following three groups: PWDs, NDs, and the general population. The PWD data were obtained from the Korea National Rehabilitation Center (KNRC) database among Korean registered disabled between 2014 and 2018, encompassing all years available from the KNRC. We aggregated 5 years of data (2014–2018) for stable model fitting because of the small population size (Additional file 1). Overall, 12,627,428 data (7,325,257 males and 5,302,171 females) were registered PWDs, among which 345,595 deaths (201,090 males and 144,505 females) were reported. The aggregated data on the general population were from the Korean Statistical Information Service between 2014 and 2018. The data included a total of 255,359,432 data (127,539,071 males and 127,820,361 females) and 1,408,559 deaths (765,722 males and 642,837 females). Subsequently, we based the data on ND as the difference between PWDs and the general population. Table 1 shows the person-years and number of deaths for each group. All utilized death data are official aggregated data, reported by collecting information at the end of the year for 1 year and subsequently estimating delayed reports for 2 years [20].

**Table 1:** Person-years and number of deaths among the study population in Korea (2014–2018)

	First age^a^ (year)	Oldest age^b^ (year)	Men		Women	
			Person-years	Death (N)	Person-years	Death (N)
General population	0	100	127,539,071	765,722	127,820,361	642,837
Non-disabled people	0	100	120,213,814	564,632	122,518,190	498,332
All people with disabilities	0	100	7,325,257	201,090	5,302,171	144,505
Physical disability	0	100	3,665,187	69,778	2,671,754	54,156
Disability of brain lesion	1	100	720,638	45,444	538,125	35,021
Visual disability	0	100	753,157	18,386	510,925	13,139
Hearing disability	0	100	767,514	28,214	652,025	21,202
Speech disability	3	95	69,977	2,447	27,585	603
Facial disfigurement	3	90	7,779	78	5,656	51
Kidney dysfunction	0	100	229,839	15,587	165,267	10,160
Cardiac dysfunction	1	90	17,870	1,303	10,574	781
Respiratory dysfunction	1	90	44,785	4,943	15,092	1,161
Hepatic dysfunction	1	85	39,802	1,462	15,599	359
Intestinal fistula/ Urinary fistula	1	100	44,461	3,145	27,671	1,754
Epilepsy	1	85	19,089	389	15,976	189
Intellectual disorder	2	95	589,240	4,879	387,970	2,584
Autistic disorder	2	60	97,613	86	17,268	23
Mental disorder	12	100	258,306	4,949	240,684	3,322

^a^Age at birth for the general population or non-disabled at the disability registration by the law in Korea for people with disabilities

^b^The oldest age in the vital statistics among the general population or non-disabled, and the Korean registered disabled by Korea National Rehabilitation Center

### Type and severity of disabilities

Enforcement Decree of the Act on Welfare of PWDs defines the following 15 disability types: physical, brain lesion, visual, hearing, and speech disabilities, facial disfigurement, kidney, cardiac, respiratory, and hepatic dysfunctions, intestinal/urinary fistula, epilepsy, intellectual disorder, autism spectrum disorder, and mental disorder [6]. After the abolition of the grading system for PWDs on July 1, 2019, the severity was classified as a degree of disability as follows: “severe disabled” and “mild disabled” [21]. The criterion for each type follows the Enforcement Rule of the same law [21].

### Analytical approach

#### Standard life table and LE of all people with disabilities

To develop a standard life table for PWDs, we employed the life table approach used by Statistics Korea [20] and the mortality extension method of Kim et al. [22]. In Step 1, we calculated the age-specific death rate (mx) using the formula below:

$${m}_{x}=\frac{the\;number\;of\;deaths\;in\;PWDs ({D}_{x})}{the\;total\;number\;of\;person-years\;in\;PWDs}$$

In Step 2, we calculated the probability of death between ages x and x + 1 (q_x_) as follows:

$${q}_{x}=\frac{the\;number\;of\;deaths}{1+(1-{a}_{x}){m}_{x}}$$

${a}_{x}$, which is called the Coale–Demeny indicator, was used to calculate the infant death rate more accurately [23]. Values of 0.05 and 0.13 for males and females were used, respectively. In Step 3, we smoothed ${q}_{x}$ using Greville’s method, and considered the best Greville order for PWDs among the 7th, 8th, and 9th. The results of each order were similar; therefore, we selected the 9th order that is commonly used. In Step 4, we extended m_x_ for the oldest-old age due to data quality limitations. Coale and Kisker’s model was fitted as an extension model in the general population. Regarding the PWDs and NDs, we determined the optimal extension model among nine functions (Additional file 2) following Kim et al. [22]. In Step 5, we recalculated the probability-adjusted q_x_ to reflect the expanded oldest-old age m_x_. In Step 6, we conducted a life table: the number of dying at age x (d_x_) was calculated as follows:

$${d}_{x}={l}_{x}\times {q}_{x}$$

Survivors ($${l}_{x}$$), $${l}_{x+1}={l}_{x}-{d}_{x}$$.

The person-years lived (${L}_{x}$), ${L}_{x}=\frac{{l}_{x}+{l}_{x+1}}{2}$.

Total number of person-years lived above age (${T}_{x}$), ${T}_{x}={\sum }_{y=x}^{\infty }{L}_{y}$.

Finally, LE (${e}_{x}$), ${e}_{x}=\frac{{T}_{x}}{{l}_{x}}$. The same method was applied to NDs and the general population. The 95% confidence interval (CI) was calculated as described in Chiang [24] as follows:

$$95\mathrm{\%\;CI\;of\;L}{\mathrm{E}}_{x}\hspace{0.17em}=\hspace{0.17em}{e}_{x} \pm 1.96\times {\mathrm{S}}_{{e}_{x}}$$

where the variances of LE ($${{S}^{2}}_{{e}_{x}})$$ and quantity p ($${{S}^{2}}_{{p}_{j}})$$ are calculated as $${{S}^{2}}_{{e}_{x}}=\frac{1}{{{l}^{2}}_{x}}\sum_{j=1}^{\infty }{{l}^{2}}_{j}[\left(1-{a}_{j}\right)+{e}_{j+1}]{{S}^{2}}_{{p}_{j}}$$, and $${{{\varvec{S}}}^{2}}_{{{\varvec{p}}}_{{\varvec{j}}}}=\frac{{{\varvec{q}}}_{{\varvec{j}}}^{2}{(1-{\varvec{q}}}_{{\varvec{j}}})}{{{\varvec{D}}}_{{\varvec{j}}}}$$, respectively.

The results were compared between the estimated life table of the general population and the official statistics from Statistics Korea to validate our model.

### Abbreviated life table and LE according to disability type and severity

After developing the standard life table for PWDs and ND, we modeled the life table based on sex, severity, and disability types. The missing age-specific m_x_ for some disability types was imputed using logistic regression between m_x_ from the standard life table and the observed m_x_ to conduct type-specific life tables [25]. Subsequently, we used the estimated m_x_ to obtain an abbreviated life table and LE for each disability type and severity, as previously described in Steps 1–6.

### HALE using the Sullivan method

The Sullivan method was widely used due to its high availability and use of prevalence data [12]. In Statistics Korea, through a survey, disability-free LE (HALE-DF) and perceived health LE (HALE-PH) were calculated as HALE [12]. HALE-DF used the following question: “Have you ever been ill from disease or accident for 2 weeks?” The average percentage of days sick for 2 weeks among those who responded was the prevalence of age x (${\pi }_{x}$) [12]. HALE-PH used a 5-point Likert scale question as follows: “How is your overall health?” Very good, good, acceptable, poor, very poor. ${\pi }_{x}$ of HALE-PH was the percentage of people who answered “It is very poor” or “poor.” [12]. We used the Social Survey [26] in Statistics Korea [27] for the NDs and the general population and the National Survey of Disabled Persons [28] for PWDs. Additionally, we used the Korea National Health Insurance System (NHIS) sample cohort database, which covers 100% of the disease claims of the Korean population [29]. It contains individual-level personal data, providing disability severity and type and medical records [29]. We obtained objective ${\pi }_{x}$ as the percentage of persons who were hospitalized for  ≥ 7 days [30]. Using each ${\pi }_{x}$, HALE was computed as follows:

$$\frac{\sum_{i=x}^{w}({L}_{i}\times \left(1-{\pi }_{x}\right))}{{l}_{x}}$$

where x is age, w is the oldest age, and ${l}_{x}$ and ${L}_{x}$ are in the life table. The 95% CI was calculated using the same method as that of LE [24]. Since some disability types had many missing values in the survey data, type-specific HALE could only be computed using NHIS (HALE-NHIS); therefore, we focused on HALE-NHIS.

## Results

Table 1 presents the distribution of registered PWDs according to disability type. Most disabilities were registered at 0 or 1 year old, except for speech disability and facial disfigurement (3 years old), intellectual disorder and autism spectrum disorder (2 years old), and mental disorder (12 years old). Among the 15 disability types, in both sexes, individuals with physical disabilities and facial disfigurement constituted the largest and smallest population portion, respectively. The number of deaths over the 5 years (2014–2018) in males associated with a speech disability, facial disfigurement, cardiac dysfunction, hepatic dysfunction, epilepsy, and autism spectrum disorder was  < 1,000, whereas that in females linked with facial disfigurement, epilepsy, and autism spectrum disorder was  < 500.

**Fig. 1:**
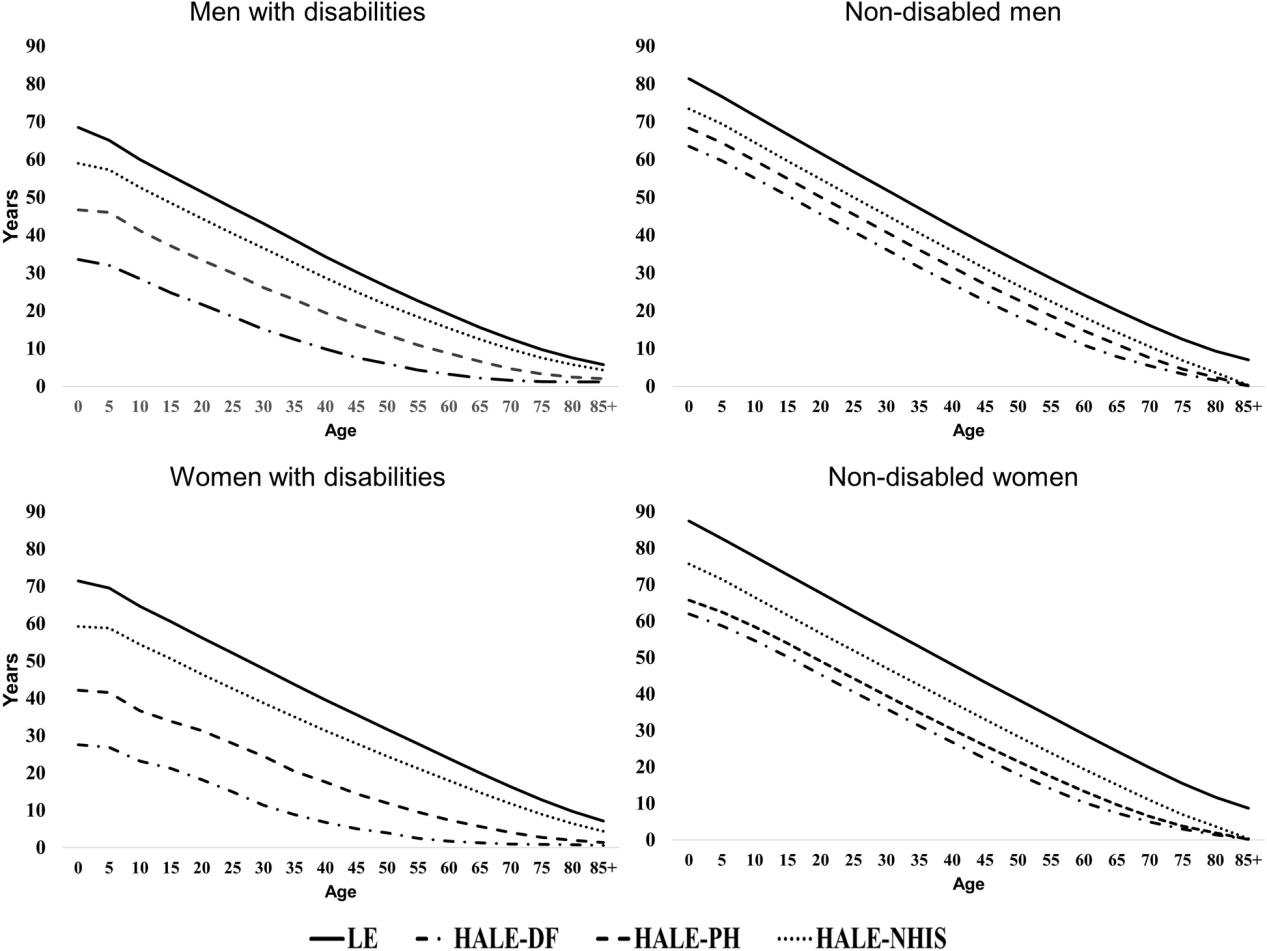
Life expectancy and healthy life expectancy by disability status, age, and sex in Korea (2014–2018). LE, life expectancy; HALE, healthy LE; HALE-DF, disability-free LE; HALE-PH, perceived health LE; HALE-NHIS, HALE using the national health insurance system database. LE and HALE were calculated using the Sullivan method

Figure 1 shows the LE and three types of HALE, between 2014 and 2018, for PWDs and NDs based on sex. The LE and HALEs of PWDs were lower than those of NDs in both sexes, although females had higher LE than males, irrespective of the disability type. HALE-DF and HALE-PH were estimated to be lower than HALE-NHIS. However, HALEs of NDs were lower than those of PWDs from age ˃70 years (Fig. 1 and Additional file 3). Therefore, we verified the accuracy of our model by comparing the estimated values using our method for the general population with the official statistics from Statistics Korea (Additional files 4 and 5).

Table 2 shows the abbreviated LE and HALE-NHIS based on the severity of disabilities and sex. In males, LEs (95% CI) at birth were 68.54 (68.48–68.60) and 81.32 (81.27–81.37) years for PWDs and NDs, respectively. In females, LEs (95% CI) at birth were 71.43 (71.37–71.49) and 87.38 (87.36–87.41) years for PWDs and NDs, respectively. LEs at birth for PWDs were lower, approximately 12.78 and 15.95 years for males and females, respectively, than those of NDs. In males, LEs (95% CI) at birth for people with severe disabilities, those with mild disabilities, and NDs were 61.53 (61.35–61.72), 73.87 (73.68–74.05), and 81.32 (81.27–81.37) years, respectively. The differences were 19.79 and 7.45 years in those with severe and mild disabilities, respectively, compared to NDs among males. In females, LEs (95% CI) at birth for people with severe disabilities, those with mild disabilities, and NDs were 62.41 (62.00–62.82), 78.25 (77.84–78.66), 87.38 (87.36–87.41) years, respectively. The differences were 24.97 and 9.13 years in those with severe and mild disabilities, respectively, compared to NDs among females. HALE-NHIS (95% CI) at birth for PWDs was 58.98 (58.96–58.99) and 59.24 (59.20–59.29) years in male and female, respectively, while that of NDs was 73.32 (73.32–73.33) and 75.58 (75.57–75.58) years in male and female, respectively. Differences in HALE-NHIS at birth for PWDs compared with NDs were 14.34 and 16.34 years for males and females, respectively. However, the differences in HALE-NHIS were greater than those of LEs between NDs and PWDs. From age 75 and 70 years for males and females, respectively, the HALE-NHIS of NDs was lower than that of PWDs. For HALE-NHIS at birth for people with mild disabilities compared to NDs, there was a difference of 7.89 and 8.07 years for males and females, respectively, and for those with severe disabilities, the differences were 22.84 and 25.86 years for males and females, respectively.

**Table 2:** Abbreviated life expectancy and HALE-NHIS database by disability, severity, and sex in Korea (2014–2018)

	Non-disabled people		People with disabilities					
			All		Mildly disabled		Severely disabled	
	LE (95% CI)	HALE-NHIS (95% CI)	LE (95% CI)	HALE-NHIS (95% CI)	LE (95% CI)	HALE-NHIS (95% CI)	LE (95% CI)	HALE-NHIS (95% CI)
Age (years)								
Male								
0	81.32 (81.27-81.37)	73.32 (73.32–73.33)	68.54 (68.48–68.60)	58.98 (58.96–58.99)	73.87 (73.68–74.05)	65.43 (65.27–65.58)	61.53 (61.35–61.72)	50.48 (50.28–50.67)
5	76.59 (76.54–76.64)	69.25 (69.25–69.26)	65.13 (65.07–65.19)	57.31 (57.31–57.32)	70.99 (70.81–71.18)	63.87 (63.82–63.93)	60.38 (60.37–60.39)	50.76 (50.71–50.81)
10	71.60 (71.55–71.65)	64.40 (64.39–64.40)	60.04 (59.98–60.10)	52.59 (52.59–52.60)	66.46 (66.27–66.64)	59.68 (59.63–59.74)	56.39 (56.38–56.40)	46.96 (46.91–47.01)
15	66.62 (66.57–66.67)	59.51 (59.50–59.51)	55.68 (55.62–55.74)	48.43 (48.42–48.44)	61.86 (61.67–62.04)	55.42 (55.38–55.47)	52.26 (52.25–52.27)	42.94 (42.89–42.98)
20	61.70 (61.64–61.75)	54.69 (54.69–54.70)	51.44 (51.38–51.50)	44.40 (44.39–44.40)	57.33 (57.15–57.52)	51.22 (51.18–51.25)	48.27 (48.26–48.28)	39.06 (39.02–39.10)
25	56.80 (56.75–56.86)	49.91 (49.90–49.91)	47.23 (47.17–47.28)	40.43 (40.42–40.43)	52.83 (52.65–53.02)	46.97 (46.94–47.01)	44.30 (44.29–44.31)	35.26 (35.21–35.30)
30	51.94 (51.89–52.00)	45.12 (45.12–45.13)	42.96 (42.90–43.02)	36.50 (36.49–36.51)	48.30 (48.11–48.49)	42.68 (42.65–42.71)	40.26 (40.25–40.27)	31.52 (31.48–31.56)
35	47.11 (47.06–47.16)	40.38 (40.38–40.39)	38.65 (38.59–38.71)	32.59 (32.59–32.60)	43.75 (43.56–43.93)	38.37 (38.35–38.40)	36.17 (36.16–36.18)	27.87 (27.83–27.91)
40	42.31 (42.26–42.36)	35.69 (35.69–35.70)	34.34 (34.28–34.40)	28.69 (28.69–28.70)	39.20 (39.01–39.38)	34.08 (34.05–34.11)	32.07 (32.06–32.07)	24.32 (24.29–24.36)
45	37.60 (37.55–37.65)	31.10 (31.09–31.10)	30.19 (30.13–30.25)	24.99 (24.98–24.99)	34.78 (34.59–34.96)	29.93 (29.91–29.96)	28.15 (28.15–28.16)	21.08 (21.04–21.11)
50	33.01 (32.95–33.06)	26.62 (26.61–26.62)	26.26 (26.20–26.32)	21.53 (21.53–21.54)	30.53 (30.34–30.71)	25.97 (25.95–25.99)	24.49 (24.49–24.50)	18.14 (18.10–18.18)
55	28.56 (28.51–28.61)	22.31 (22.31–22.32)	22.55 (22.49–22.61)	18.32 (18.31–18.33)	26.47 (26.28–26.65)	22.24 (22.21–22.26)	21.08 (21.07–21.09)	15.47 (15.44–15.51)
60	24.24 (24.19–24.30)	18.18 (18.18–18.19)	18.99 (18.93–19.05)	15.28 (15.28–15.29)	22.55 (22.36–22.73)	18.70 (18.67–18.72)	17.83 (17.82–17.83)	12.98 (12.94–13.01)
65	20.06 (20.00–20.11)	14.20 (14.20–14.21)	15.65 (15.59–15.71)	12.47 (12.46–12.47)	18.82 (18.64–19.01)	15.39 (15.37–15.42)	14.79 (14.79–14.80)	10.69 (10.65–10.72)
70	16.06 (16.00–16.11)	10.40 (10.39–10.40)	12.49 (12.43–12.55)	9.83 (9.82–9.83)	15.30 (15.11–15.48)	12.29 (12.27–12.31)	11.95 (11.94–11.95)	8.54 (8.51–8.57)
75	12.39 (12.34–12.44)	6.85 (6.84–6.85)	9.74 (9.68–9.80)	7.55 (7.55–7.56)	12.19 (12.00–12.37)	9.57 (9.55–9.59)	9.52 (9.51–9.52)	6.71 (6.68–6.74)
80	9.32 (9.27–9.38)	3.60 (3.60–3.60)	7.49 (7.43–7.55)	5.70 (5.69–5.70)	9.60 (9.42–9.79)	7.30 (7.29–7.32)	7.58 (7.58–7.59)	5.25 (5.23–5.28)
85+	7.01 (6.96–7.07)	0.35 (0.35–0.35)	5.70 (5.64–5.76)	4.26 (4.26–4.27)	7.54 (7.35–7.72)	5.49 (5.48–5.49)	6.10 (6.10–6.10)	4.12 (4.11–4.13)
Female								
0	87.38 (87.36–87.41)	75.58 (75.57–75.58)	71.43 (71.37–71.49)	59.24 (59.20–59.29)	78.25 (77.84–78.66)	67.51 (67.34–67.68)	62.41 (62.00–62.82)	49.72 (49.33–50.11)
5	82.62 (82.60–82.65)	71.41 (71.41–71.41)	69.45 (69.42–69.49)	58.78 (58.78–58.79)	76.49 (76.08–76.90)	66.51 (66.44–66.58)	63.38 (63.36–63.39)	51.89 (51.83–51.96)
10	77.63 (77.61–77.65)	66.52 (66.52–66.53)	64.58 (64.55–64.62)	54.43 (54.43–54.44)	72.07 (71.66–72.48)	62.43 (62.38–62.49)	59.89 (59.88–59.91)	48.70 (48.64–48.76)
15	72.65 (72.63–72.67)	61.61 (61.61–61.61)	60.47 (60.43–60.50)	50.53 (50.53–50.54)	67.50 (67.10–67.91)	58.23 (58.18–58.28)	56.06 (56.05–56.07)	44.99 (44.94–45.04)
20	67.70 (67.67–67.72)	56.75 (56.74–56.75)	56.19 (56.15–56.22)	46.45 (46.44–46.45)	62.85 (62.44–63.25)	53.86 (53.81–53.90)	52.01 (52.00–52.02)	41.05 (41.01–41.10)
25	62.76 (62.74–62.78)	51.89 (51.89–51.90)	52.08 (52.05–52.12)	42.55 (42.54–42.55)	58.30 (57.89–58.71)	49.55 (49.51–49.58)	48.19 (48.18–48.20)	37.35 (37.30–37.39)
30	57.84 (57.82–57.87)	47.09 (47.09–47.10)	47.89 (47.86–47.92)	38.69 (38.69–38.70)	53.70 (53.30–54.11)	45.29 (45.26–45.32)	44.24 (44.23–44.25)	33.68 (33.64–33.72)
35	52.96 (52.93–52.98)	42.41 (42.41–42.42)	43.74 (43.71–43.78)	35.00 (35.00–35.00)	49.14 (48.74–49.55)	41.12 (41.10–41.15)	40.36 (40.35–40.37)	30.20 (30.16–30.24)
40	48.09 (48.07–48.11)	37.72 (37.72–37.72)	39.65 (39.61–39.68)	31.36 (31.36–31.37)	44.63 (44.22–45.03)	36.97 (36.94–36.99)	36.54 (36.53–36.54)	26.83 (26.80–26.86)
45	43.25 (43.23–43.28)	33.01 (33.01–33.02)	35.64 (35.61–35.68)	27.86 (27.86–27.86)	40.18 (39.77–40.59)	32.87 (32.85–32.89)	32.82 (32.82–32.83)	23.66 (23.63–23.69)
50	38.45 (38.43–38.48)	28.37 (28.36–28.37)	31.70 (31.67–31.73)	24.47 (24.46–24.47)	35.79 (35.38–36.19)	28.86 (28.84–28.88)	29.19 (29.18–29.19)	20.65 (20.62–20.68)
55	33.69 (33.67–33.72)	23.84 (23.83–23.84)	27.79 (27.76–27.82)	21.20 (21.20–21.20)	31.43 (31.02–31.84)	25.00 (24.98–25.02)	25.58 (25.58–25.59)	17.81 (17.78–17.84)
60	28.97 (28.94–28.99)	19.41 (19.41–19.42)	23.88 (23.84–23.91)	17.97 (17.97–17.97)	27.09 (26.68–27.50)	21.24 (21.22–21.25)	21.96 (21.96–21.97)	15.04 (15.01–15.07)
65	24.30 (24.28–24.33)	15.07 (15.07–15.07)	20.03 (19.99–20.06)	14.80 (14.80–14.80)	22.82 (22.41–23.23)	17.58 (17.57–17.60)	18.41 (18.41–18.42)	12.37 (12.34–12.39)
70	19.75 (19.72–19.77)	10.89 (10.89–10.89)	16.28 (16.25–16.31)	11.73 (11.72–11.73)	18.66 (18.25–19.07)	14.06 (14.05–14.08)	14.97 (14.97–14.98)	9.82 (9.80–9.84)
75	15.45 (15.43–15.47)	6.90 (6.90–6.91)	12.81 (12.78–12.84)	8.88 (8.88–8.88)	14.78 (14.37–15.18)	10.80 (10.79–10.81)	11.84 (11.84–11.85)	7.55 (7.53–7.57)
80	11.65 (11.62–11.67)	3.73 (3.73–3.73)	9.73 (9.70–9.76)	6.43 (6.42–6.43)	11.31 (10.90–11.72)	7.98 (7.97–7.99)	9.14 (9.14–9.15)	5.72 (5.70–5.73)
85+	8.65 (8.62–8.67)	0.35 (0.34–0.35)	7.13 (7.10–7.17)	4.38 (4.38–4.38)	8.42 (8.01–8.83)	5.72 (5.71–5.72)	6.97 (6.97–6.97)	4.31 (4.31–4.32)

*LE* Life expectancy, *HALE-NHIS* Healthy life expectancy using the national health insurance system database, *CI* Confidence interval

**Fig. 2:**
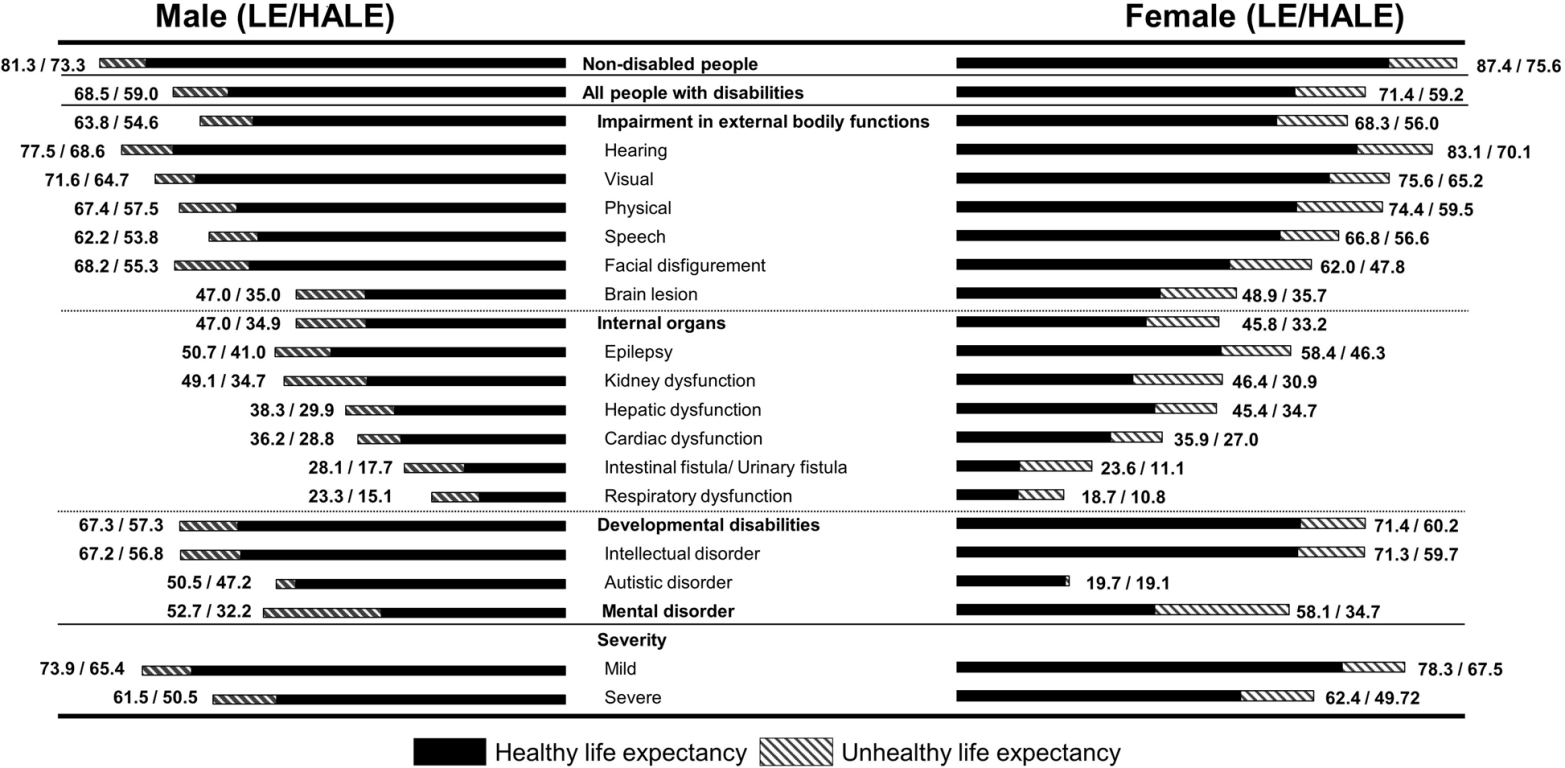
Summary of life expectancy and healthy life expectancy using the National Health Insurance System database  of  non-disabled and people with disabilities according to type and severity. The unhealthy life expectancy was the difference between life expectancy and healthy life expectancy

Figure 2 describes type-specific LE and HALE-NHIS at birth or first age, between 2014 and 2018, for PWDs and NDs. For both sexes, people with hearing disabilities had the longest LE and HALE-NHIS at first age, 77.5 and 68.6 years in males and 83.1 and 70.1 years in females. However, LE/HALE-NHIS (years) at the first age of people with internal organs disabilities was lower as follows: epilepsy (50.7/41.0 in males and 58.4/46.3 in females), kidney dysfunction (49.1/34.7 in males and 46.4/30.9 in females), hepatic dysfunction (38.3/29.9 in males and 45.4/34.7 in females), cardiac dysfunction (36.2/28.8 in males and 35.9/27.0 in females), intestinal/urinary fistula (28.1/17.7 in males and 23.6 /11.1 in females), and respiratory dysfunction (23.3/15.1 in males and 18.7/10.8 in females). People with autism spectrum disorder and those with mental disorders had the smallest (3.3 in males and 0.6 in females) and largest (20.5 in males and 23.4 in females) differences, respectively. Furthermore, the gap between LE and HALE-NHIS in females was greater than that in males, except for respiratory dysfunction and an autism spectrum disorder. Additional files 6 and 7 summarizes the LE and HALE-NHIS per 5 years of age by sex and disability type, respectively.

## Discussion

According to the data gathered and analyzed between 2014 and 2018, we found that males with disabilities had lower LE/HALE-NHIS at the first age than females (males and females were 68.54/58.98 and 71.43/59.24 years, respectively). For both sexes, the disability type with the highest and lowest LE and HALE-NHIS were hearing disability and respiratory dysfunction, respectively. Furthermore, autism spectrum disorder and mental disorder had the smallest and largest gap between LE and HALE-NHIS, respectively.

In this study, the LE at birth for PWDs was lower than that of NDs. The level of LE for PWDs (2014–2018) was similar to that of the Korean general population from 1980 to 1990 (Males: 68.4 years in 1992, Females: 71.5 years in 1982) [31]. Additionally, the level of LE at birth for PWDs (2014–2018) was similar to that of Russia in 2019 (68.2 years) for males [32] and India in 2019 (72.2 years) for females [32]. Males with disabilities had a lower LE at the first age than females. Another study on PWDs in Korea showed comparable results, which were 66.3 and 70.2 years for males and females in 2017, respectively [31].

We observed the LE and HALE-NHIS at the first age of people with hearing disabilities and those with respiratory dysfunction were the highest and lowest, respectively. Differences were observed among the disability types. Compared with previous studies that calculated LE using NHIS in Korea, the LE of PWDs with hearing disabilities was similar at 75.1 (our result was 77.5 years) and 81.3 (our result was 83.1 years) years for males and females, respectively; those of respiratory disability were also similar at 20.7 (our result was 23.3 years) and 17.1 (our result was 18.7 years) years for males and females, respectively [17]. The average age (65.8 years) and age at death (80.3 years) of PWDs with hearing disabilities were higher than those of PWDs with other disabilities; therefore, the LE tended to be estimated as high [33]. Overall, the LE reported in our study was estimated to be higher than that reported by Bahk et al. [17]. Although it is difficult to assess the accuracy of this estimation because there were no official statistics on PWDs in Korea, we may consider several possibilities regarding the difference between the studies. First, these could be attributed to the data source. In our study, we used official data directly, which ensured its accuracy and reliability. In the study by Bahk et al. [17], mortality data were linked to NHIS data; thus, it may differ from the official data [17]. Second, modeling was employed in this study. We developed a standard model for PWDs, whereas Bahk et al. [17] adopted a model that was suitable for the general population. Third, we aggregated data over a 5-year period (2014–2018), whereas Bahk et al. aggregated data over a 10-year period (2008–2017); thus, the deaths reported in our study reflected recent mortality trends. Thus, considering the known improvements in mortality rates, it is possible that our mortality data provided lower estimates and our LE data provided higher estimates than Bahk et al. [17].”

Notably, our results can be compared to those of other countries. Similarly, in the case of patients with traumatic brain injury who had some walking ability in the United States, LE was estimated to be 50 and 55 years for males and females, respectively [13]. Conversely, the results of other disability types, such as autism spectrum disorder, differed from those of previous studies because the classification of disability in Korea encompasses various disability states while targeting specific disease groups in other countries. For instance, the LE of 5-year-old males with autism spectrum disorder was 52.0 and 62.0 years in our study and that of Shavelle and Strauss [34], respectively, with a 10-year difference, whereas it was 47.0 and 62.5 years in our study and that of Shavelle and Strauss [34], respectively, for females, with a difference of 15.5 years (Additional file 6). The LE of females with an autism spectrum disorder in our study was significantly low because person-years and the number of deaths were relatively small for a stable fitting model. However, the studies on HALE were more limited. Furthermore, there are no type-specific studies in Korea, and it is difficult to compare the HALE in other countries because the targeted PWDs were specific age groups, and the definition of prevalence was inconsistent [14, 16, 18, 35].

The differences between LE and HALE estimates among people with severe disabilities were greater than that of those with mild disabilities. However, the difference indicates that when considering lower LE, the HALE, calculated by the rate of hospitalization for more than seven days, for PWDs with severe disabilities was lower than that for PWDs with mild disabilities. Therefore, the severity of the disability could affect LE and HALE estimates related to hospitalization prevalence. A similar result was obtained in a study from Brazil. In a study using the City of Campinas Health Survey for Brazilian older adults, the HALE of people aged 60 years with a mild or moderate functional disability was 13.5 and 14.9 years in males and females, respectively, whereas that for those with a severe functional disability was 17.5 and 20.0 years in males and females, respectively [19].

Regarding methodologies, this study contributed important advances to constructing a suitable life table model for PWDs. Previous studies in Korea calculated LE using a single general life table model and targeted the entire population of PWDs [27, 36]. In our study, we attempted to develop an optimal life table model for the entire population of PWDs and include each disability type. Therefore, we conducted a similar process to that of the general population to evaluate our method and compare official statistics and estimated values. Furthermore, we measured age-specific LE across all ages despite the studies of other countries being confined to the abbreviated life tables.

We found that the different HALEs were estimated according to the prevalence measures, which is consistent with Kang et al. [30] HALE-NHIS was higher than HALE-DF and HALE-PH, and the gap between PWDs and NDs for HALE-NHIS did not differ from that of LE. Additionally, the gap between groups depended on variations in mortality for LE and prevalence for HALE. The crude death rate of PWDs was four times that of the general population [33], whereas the number of inpatient days per person was approximately 2.5 times [37]. Moreover, the inpatient days may be underestimated due to the high unmet healthcare needs (UHCNs) among PWDs [4].

From age ˃70 years (75 and 70 years old in males and females, respectively), the HALE-NHIS of NDs was lower than that of PWDs. However, the reverse phenomenon may occur in the older age group because PWDs had more opportunities for medical care services based on the Korean disability policy, which was aimed at strengthening career support systems for stable income and enhancing the accessibility of healthcare services for PWDs [5]. The proportion of UHCNs among PWDs was lower than that of NDs in 2015 among older adults [4]. Therefore, improving welfare policies for PWDs could affect the HALE-NHIS of the older PWDs by decreasing the proportion of UHCNs for PWDs, whereas that of the older NDs in the welfare blind spots may be relatively lowered.

This study had some limitations. First, the original KNRC data for each year were insufficient. Therefore, we aggregated all available data; this method is used in Norway, with a small population [38] (approximately 5,370,000 in 2020) [39]. Although the data from NHIS [17] could be used to obtain sufficient samples, the data could not reflect the actual number of registered PWDs because the data was established at the beginning of each year. For example, Bahk et al. [17] presented 35,660,309 person-years (2008–2017), whereas the official number was 24,776,890. Second, the data for each disability type were also insufficient. Therefore, obtaining the number of deaths for all ages was challenging. However, to overcome this limitation, we requested more data for additional years but could not receive it; therefore, we fitted the non-linear regression model to impute missing values. Finally, LE in our study was period LE, which assumed that the death rates applied equally throughout the remainder of the person’s life [40]. Because cohort refers to a group of people with the same birth year, a cohort LE may be appropriate for our data based on registered PWDs with different registration years [40]. Although period LE tends to be lower than cohort LE, the differences are historically comparable [40]. Despite these limitations, our study is the first in Korea to develop the characterizing life table model and estimate LE and HALEs based on the type of disability by sex. Therefore, these findings present the health and welfare risks facing PWDs in Korea.

## Conclusion

In this study, males with disabilities had a shorter gap between LE and HALEs than females, except for those with severe disabilities. Furthermore, there were variabilities according to the disability types. Therefore, future studies based on each disability type should be conducted to strengthen the monitoring and the health system infrastructures for each disability type.

### Supplementary Information

**Additional file 1.** Number of registered people with disability in Korea by year (2014–2018).

**Additional file 2.** Function for estimating mortality of older age.

**Additional file 3.** Abbreviated healthy life expectancy using the Sullivan method through survey data by disability, severity, and sex in Korea (2014–2018).

**Additional file 4.** Abbreviated life table of standard model for Korean general population by sex (2014–2018).

**Additional file 5.** Comparison of estimated healthy life expectancy (2014–2018) from this study and the official statistics in 2016 from the Korean Statistical Information Service among Korean general population.

**Additional file 6.** Abbreviated life expectancy by type of disability and sex in Korea (2014–2018).

**Additional file 7.** Abbreviated healthy life expectancy using the National Health Insurance System database by type of disability and sex in Korea (2014–2018).

## Data Availability

The annual population for PWDs, NDs, and the general population by age and sex can get at KOSIS [https://kosis.kr/eng/]. The number of annual death for NDs and the general population by age and sex can get at KOSIS [https://kosis.kr/eng/]. The annual number of death and the annual prevalence of persons who were hospitalized for more than seven days for PWDs can get at KNRC. However, KNRC data were used under license for the current study and are not publicly available. The availability of KNRC data should be confirmed at openinfo@korea.kr (Representative email of Korea open information portal). The data on the annual prevalence of persons who were hospitalized for more than seven days for NDs and the general population can get from the NHIS sample cohort database at [https://nhiss.nhis.or.kr/bd/ab/bdaba000eng.do]. For using the NHIS sample cohort database, there is a need for reasonable requests, IRB admission, and permission from NHIS. The data about the percentage of days sick for two weeks of NDs and the general population can get from the Social survey at [https://mdis.kostat.go.kr/index.do]. Those of PWD can get from the National Survey of Disabled Persons at [https://data.kihasa.re.kr/kihasa/kor/contents/ContentsList.html].

## Abbreviations

LE: Life expectancy
HALE: Healthy life expectancy
PWDs: People with disabilities
ND: Non-disabled people
KNRC: Korea National Rehabilitation Center
HALE-DF: Disability-free life expectancy
HALE-PH: Perceived health life expectancy
NHIS: Korea National Health Insurance System
HALE-NHIS: Healthy life expectancy using the Korean National Health Insurance System sample cohort database
