# Supplementary material for: Life expectancy and healthy life expectancy of Korean registered disabled by disability type in 2014–2018: Korea National Rehabilitation Center database
Source: BMC Public Health. 2023 Sep 8;23:1750. doi: 10.1186/s12889-023-16682-9 (PMC10485940; doi:10.1186/s12889-023-16682-9)
Supplement: Supplementary file 1 — Additional file 1. Number of registered people with disability in Korea by year (2014–2018). [file 12889_2023_16682_MOESM1_ESM.docx]

Additional file 1. Number of registered people with disability in Korea by year (2014–2018)

|  | Male | | | | | | Female | | | | | |
| --- | --- | --- | --- | --- | --- | --- | --- | --- | --- | --- | --- | --- |
|  | Total | 2014 | 2015 | 2016 | 2017 | 2018 | Total | 2014 | 2015 | 2016 | 2017 | 2018 |
| All people with disability | **7,325,257** | **1,448,878** | **1,446,943** | **1,457,588** | **1,475,550** | **1,496,298** | **5,302,172** | **1,045,582** | **1,043,463** | **1,053,462** | **1,070,087** | **1,089,578** |
| Physical | 3,665,187 | 749,259 | 741,089 | 732,667 | 725,384 | 716,788 | 2,671,754 | 546,349 | 540,408 | 534,507 | 528,746 | 521,744 |
| Brain lesion | 720,638 | 143,931 | 143,637 | 143,359 | 144,706 | 145,005 | 538,125 | 107,612 | 107,225 | 107,097 | 108,113 | 108,078 |
| Visual | 753,157 | 150,843 | 150,883 | 150,752 | 150,364 | 150,315 | 510,925 | 101,982 | 101,991 | 102,042 | 102,268 | 102,642 |
| Hearing | 767,514 | 138,356 | 136,850 | 147,749 | 162,319 | 182,240 | 652,026 | 114,423 | 113,484 | 124,093 | 139,684 | 160,342 |
| Speech | 69,977 | 13,095 | 13,483 | 13,900 | 14,590 | 14,909 | 27,585 | 5,180 | 5,330 | 5,509 | 5,731 | 5,835 |
| Facial disfigurement | 7,779 | 1,557 | 1,555 | 1,549 | 1,557 | 1,561 | 5,656 | 1,132 | 1,130 | 1,131 | 1,135 | 1,128 |
| Kidney dysfunction | 229,839 | 40,650 | 43,132 | 45,769 | 48,768 | 51,520 | 165,267 | 29,784 | 31,336 | 32,981 | 34,794 | 36,372 |
| Cardiac dysfunction | 17,870 | 4,000 | 3,646 | 3,450 | 3,405 | 3,369 | 10,574 | 2,401 | 2,187 | 2,057 | 1,994 | 1,935 |
| Respiratory dysfunction | 44,785 | 9,392 | 8,990 | 8,822 | 8,819 | 8,762 | 15,092 | 3,053 | 3,043 | 3,009 | 2,988 | 2,999 |
| Hepatic dysfunction | 39,802 | 7,000 | 7,445 | 7,938 | 8,494 | 8,925 | 15,599 | 2,668 | 2,879 | 3,104 | 3,349 | 3,599 |
| Intestinal fistula/Urinary fistula | 44,461 | 8,513 | 8,683 | 8,876 | 9,098 | 9,291 | 27,671 | 5,354 | 5,433 | 5,528 | 5,620 | 5,736 |
| Epilepsy | 19,089 | 3,876 | 3,858 | 3,775 | 3,763 | 3,817 | 15,976 | 3,208 | 3,211 | 3,181 | 3,172 | 3,204 |
| Intellectual disorder | 589,240 | 111,183 | 114,419 | 117,725 | 121,167 | 124,746 | 387,970 | 73,172 | 75,333 | 77,558 | 79,736 | 82,171 |
| Autism spectrum disorder | 97,613 | 16,629 | 17,964 | 19,419 | 20,972 | 22,629 | 17,268 | 2,895 | 3,139 | 3,434 | 3,726 | 4,074 |
| Mental disorder | 258,306 | 50,594 | 51,309 | 51,838 | 52,144 | 52,421 | 240,684 | 46,369 | 47,334 | 48,231 | 49,031 | 49,719 |
