# Supplementary material for: Life expectancy and healthy life expectancy of Korean registered disabled by disability type in 2014–2018: Korea National Rehabilitation Center database
Source: BMC Public Health. 2023 Sep 8;23:1750. doi: 10.1186/s12889-023-16682-9 (PMC10485940; doi:10.1186/s12889-023-16682-9)
Supplement: Supplementary file 2 — Additional file 2. Function for estimating mortality of older age. [file 12889_2023_16682_MOESM2_ESM.docx]

Additional file 2. Function for estimating mortality of older age

| Function | Formula | Number of parameters |
| --- | --- | --- |
| Gompertz | $m_{x}=be^{ax}$ | 2 |
| Makeham | $m_{x}=c+be^{ax}$ | 3 |
| Coale and Kisker | $k_{x}=a+bx$ | 3 |
| Himes | ${logit m}_{x}=a+bx$ | 2 |
| Heligman–Pollard | ${logit q}_{x}=a+bx$ | 4 |
| Weibull | $m_{x}=bx^{a}$ | 2 |
| Shifted Weibull | $m_{x}=c+bx^{a}$ | 3 |
| Quadratic function | $m_{x}=a+bx+cx^{2}$ | 3 |
| Cubic function | $m_{x}=a+bx+cx^{2}+dx^{3}$ | 4 |

$m_{x}$*,* age-specific death rate between ages x and x+1; $q_{x},$ probability of dying between ages x and x+1; $k_{x}=\log(\frac{m_{x}}{m_{x-1}})$
