# Supplementary material for: Life expectancy and healthy life expectancy of Korean registered disabled by disability type in 2014–2018: Korea National Rehabilitation Center database
Source: BMC Public Health. 2023 Sep 8;23:1750. doi: 10.1186/s12889-023-16682-9 (PMC10485940; doi:10.1186/s12889-023-16682-9)
Supplement: Supplementary file 3 — Additional file 3. Abbreviated healthy life expectancy using the Sullivan method through survey data by disability, severity, and sex in Korea (2014–2018). [file 12889_2023_16682_MOESM3_ESM.docx]

Additional file 3. Abbreviated healthy life expectancy using the Sullivan method through survey data by disability, severity, and sex in Korea (2014–2018)

|  | Non-disabled people | | People with disabilities | | | | | |
| --- | --- | --- | --- | --- | --- | --- | --- | --- |
|  |  |  | All | | Mild disabled | | Severe disabled | |
|  | DF^a^ | PH^b^ | DF^a^ | PH^b^ | DF^a^ | PH^b^ | DF^a^ | PH^b^ |
| Male |  |  |  |  |  |  |  |  |
| Age (years) | | | | | | | | |
| 0 | 63.4 | 68.3 | 33.6 | 46.6 | 39.1 | 53.6 | 27.0 | 38.7 |
| 5 | 59.6 | 64.4 | 32.0 | 46.0 | 37.4 | 50.6 | 27.8 | 39.5 |
| 10 | 55.1 | 59.8 | 28.5 | 41.1 | 34.4 | 46.2 | 24.8 | 35.3 |
| 15 | 50.4 | 54.9 | 24.7 | 37.1 | 31.4 | 42.9 | 21.5 | 31.3 |
| 20 | 45.6 | 50.1 | 21.7 | 33.4 | 27.1 | 39.5 | 18.2 | 27.7 |
| 25 | 40.9 | 45.5 | 18.4 | 30.0 | 23.6 | 35.6 | 15.3 | 24.5 |
| 30 | 36.1 | 40.7 | 15.0 | 26.0 | 19.7 | 31.3 | 11.9 | 20.7 |
| 35 | 31.5 | 36.1 | 12.4 | 22.9 | 16.3 | 27.9 | 9.4 | 17.6 |
| 40 | 27.0 | 31.5 | 9.9 | 19.5 | 13.3 | 24.1 | 7.5 | 14.4 |
| 45 | 22.6 | 27.0 | 7.6 | 16.3 | 10.2 | 20.0 | 5.8 | 12.4 |
| 50 | 18.5 | 22.8 | 6.0 | 13.6 | 7.8 | 16.8 | 4.9 | 10.2 |
| 55 | 14.5 | 18.7 | 4.3 | 10.9 | 5.5 | 13.4 | 3.7 | 8.2 |
| 60 | 10.9 | 14.7 | 3.1 | 8.7 | 3.9 | 10.7 | 2.7 | 6.6 |
| 65 | 7.9 | 11.1 | 2.2 | 6.6 | 2.7 | 8.2 | 2.0 | 4.9 |
| 70 | 5.4 | 7.5 | 1.6 | 4.7 | 1.8 | 5.9 | 1.1 | 3.5 |
| 75 | 3.2 | 4.5 | 1.2 | 3.3 | 1.4 | 4.1 | 0.8 | 2.8 |
| 80 | 1.5 | 2.3 | 1.1 | 2.5 | 1.1 | 2.8 | 0.6 | 2.6 |
| 85+ | 0.2 | 0.2 | 1.2 | 2.0 | 0.8 | 2.0 | 0.4 | 2.6 |
| Female |  |  |  |  |  |  |  |  |
| 0 | 62.0 | 65.7 | 27.6 | 42.1 | 39.5 | 45.6 | 24.7 | 36.4 |
| 5 | 58.7 | 62.4 | 26.9 | 41.6 | 36.9 | 44.1 | 25.6 | 37.0 |
| 10 | 54.7 | 58.4 | 23.2 | 36.6 | 32.5 | 41.1 | 21.9 | 32.9 |
| 15 | 50.1 | 53.8 | 21.2 | 33.8 | 28.2 | 37.8 | 19.5 | 30.2 |
| 20 | 45.4 | 49.0 | 18.2 | 31.3 | 24.1 | 34.0 | 16.8 | 27.9 |
| 25 | 40.6 | 44.3 | 14.9 | 27.9 | 20.5 | 29.7 | 13.8 | 24.8 |
| 30 | 35.9 | 39.5 | 11.3 | 24.5 | 18.0 | 25.6 | 9.9 | 21.4 |
| 35 | 31.3 | 34.8 | 8.9 | 20.4 | 13.8 | 22.4 | 7.6 | 17.5 |
| 40 | 26.7 | 30.3 | 6.8 | 17.6 | 10.5 | 19.8 | 6.0 | 14.6 |
| 45 | 22.3 | 25.8 | 5.1 | 14.4 | 7.8 | 16.5 | 4.8 | 11.7 |
| 50 | 17.9 | 21.5 | 3.9 | 12.0 | 5.0 | 13.7 | 3.7 | 9.5 |
| 55 | 13.9 | 17.3 | 2.5 | 9.6 | 3.3 | 11.1 | 3.0 | 7.2 |
| 60 | 10.3 | 13.3 | 1.7 | 7.3 | 2.0 | 8.6 | 2.2 | 5.3 |
| 65 | 7.3 | 9.7 | 1.3 | 5.6 | 1.3 | 6.6 | 1.8 | 4.3 |
| 70 | 4.9 | 6.4 | 0.9 | 4.0 | 1.0 | 4.7 | 1.1 | 3.1 |
| 75 | 2.9 | 3.8 | 0.8 | 2.7 | 0.7 | 3.1 | 1.0 | 2.4 |
| 80 | 1.4 | 1.9 | 0.8 | 2.0 | 0.7 | 2.2 | 1.0 | 2.0 |
| 85+ | 0.2 | 0.2 | 0.6 | 1.3 | 0.5 | 1.5 | 1.3 | 1.8 |

^a^Disability-free (DF) life expectancy

^b^Perceived health (PH) life expectancy
