# Supplementary material for: Life expectancy and healthy life expectancy of Korean registered disabled by disability type in 2014–2018: Korea National Rehabilitation Center database
Source: BMC Public Health. 2023 Sep 8;23:1750. doi: 10.1186/s12889-023-16682-9 (PMC10485940; doi:10.1186/s12889-023-16682-9)
Supplement: Supplementary file 4 — Additional file 4. Abbreviated life table of standard model for Korean general population by sex (2014–2018). [file 12889_2023_16682_MOESM4_ESM.docx]

Additional file 4. Abbreviated life table of standard model for Korean general population by sex (2014–2018)

| Age (x) | qx | lx | dx | Lx | Tx | Ex | Ex16 |
| --- | --- | --- | --- | --- | --- | --- | --- |
| Male | | | | | | | |
| 0 | 0.0031 | 100,000 | 310 | 99702 | 7938253 | 79.4 | 79.3 |
| 1 | 0.0002 | 99,690 | 23 | 99845 | 7838551 | 78.6 | 78.5 |
| 5 | 0.0001 | 99,667 | 11 | 99667 | 7439685 | 74.6 | 74.6 |
| 10 | 0.0001 | 99,641 | 8 | 99637 | 6941382 | 69.7 | 69.6 |
| 15 | 0.0002 | 99,592 | 18 | 99583 | 6443285 | 64.7 | 64.7 |
| 20 | 0.0004 | 99,456 | 35 | 99439 | 5945622 | 59.8 | 59.8 |
| 25 | 0.0005 | 99,251 | 50 | 99226 | 5448822 | 54.9 | 54.9 |
| 30 | 0.0007 | 98,971 | 66 | 98938 | 4953239 | 50.0 | 50.0 |
| 35 | 0.0008 | 98,612 | 83 | 98570 | 4459251 | 45.2 | 45.2 |
| 40 | 0.0013 | 98,114 | 130 | 98049 | 3967347 | 40.4 | 40.4 |
| 45 | 0.0021 | 97,332 | 207 | 97229 | 3478589 | 35.7 | 35.7 |
| 50 | 0.0035 | 96,058 | 338 | 95889 | 2994860 | 31.2 | 31.1 |
| 55 | 0.0052 | 94,070 | 490 | 93825 | 2519242 | 26.8 | 26.7 |
| 60 | 0.0075 | 91,245 | 687 | 90902 | 2055570 | 22.5 | 22.5 |
| 65 | 0.0113 | 87,267 | 986 | 86775 | 1608719 | 18.4 | 18.4 |
| 70 | 0.0179 | 81,503 | 1462 | 80771 | 1185919 | 14.6 | 14.5 |
| 75 | 0.0333 | 72,443 | 2415 | 71235 | 799207 | 11.0 | 11.0 |
| 80 | 0.0608 | 58,111 | 3534 | 56344 | 470552 | 8.1 | 8.1 |
| 85 | 0.1048 | 38,909 | 4076 | 36871 | 226666 | 5.8 | 5.8 |
| 90 | 0.1636 | 19,613 | 3208 | 18009 | 81833 | 4.2 | 4.1 |
| 95 | 0.2326 | 6,818 | 1586 | 6025 | 19036 | 2.8 | 2.9 |
| 100 | 0.3124 | 1,471 | 460 | 736 | 736 | 0.5 | 2.1 |
| Female | | | | | | | |
| 0 | 0.0026 | 100000 | 259 | 99752 | 8575685 | 85.8 | 85.4 |
| 1 | 0.0002 | 99741 | 21 | 99871 | 8475933 | 85.0 | 84.6 |
| 5 | 0.0001 | 99720 | 8 | 99723 | 8076883 | 81.0 | 80.7 |
| 10 | 0.0001 | 99705 | 6 | 99701 | 7578287 | 76.0 | 75.7 |
| 15 | 0.0001 | 99668 | 12 | 99662 | 7079848 | 71.0 | 70.7 |
| 20 | 0.0002 | 99589 | 21 | 99578 | 6581690 | 66.1 | 65.8 |
| 25 | 0.0003 | 99476 | 27 | 99462 | 6084016 | 61.2 | 60.9 |
| 30 | 0.0004 | 99325 | 37 | 99306 | 5587000 | 56.2 | 55.9 |
| 35 | 0.0005 | 99104 | 52 | 99077 | 5090898 | 51.4 | 51.1 |
| 40 | 0.0007 | 98811 | 69 | 98777 | 4596079 | 46.5 | 46.2 |
| 45 | 0.0010 | 98419 | 94 | 98372 | 4102956 | 41.7 | 41.4 |
| 50 | 0.0014 | 97871 | 134 | 97804 | 3612149 | 36.9 | 36.6 |
| 55 | 0.0018 | 97121 | 178 | 97032 | 3124586 | 32.2 | 31.9 |
| 60 | 0.0026 | 96101 | 251 | 95975 | 2641398 | 27.5 | 27.2 |
| 65 | 0.0041 | 94613 | 385 | 94420 | 2164369 | 22.9 | 22.6 |
| 70 | 0.0074 | 92188 | 680 | 91848 | 1696827 | 18.4 | 18.1 |
| 75 | 0.0154 | 87642 | 1352 | 86966 | 1246004 | 14.2 | 13.9 |
| 80 | 0.0326 | 78712 | 2566 | 77429 | 827824.5 | 10.5 | 10.2 |
| 85 | 0.0678 | 62494 | 4239 | 60374 | 471384.3 | 7.5 | 7.1 |
| 90 | 0.1114 | 40090 | 4468 | 37856 | 214165 | 5.3 | 4.9 |
| 95 | 0.1644 | 19760 | 3249 | 18136 | 66793.21 | 3.4 | 3.3 |
| 100 | 0.2270 | 6936 | 1575 | 3468 | 3468.096 | 0.5 | 2.3 |

qx, probability of dying between ages x to x+1; lx, survivors to ages x; dx, number of dying between ages x to x+1; Lx, person-years lived between ages x to x+1; Tx, total number of person-years lived above age x; Ex, life expectancy at age x; Ex16 is official statistics on life expectancy in 2016 from Korean Statistical Information Service
