# Supplementary material for: Life expectancy and healthy life expectancy of Korean registered disabled by disability type in 2014–2018: Korea National Rehabilitation Center database
Source: BMC Public Health. 2023 Sep 8;23:1750. doi: 10.1186/s12889-023-16682-9 (PMC10485940; doi:10.1186/s12889-023-16682-9)
Supplement: Supplementary file 5 — Additional file 5. Comparison of estimated healthy life expectancy (2014–2018) from this study and the official statistics in 2016 from the Korean Statistical Information Service among Korean general population. [file 12889_2023_16682_MOESM5_ESM.docx]

Additional file 5. Comparison of estimated healthy life expectancy (2014–2018) from this study and the official statistics in 2016 from the Korean Statistical Information Service among Korean general population

| Age (years) | Men | | | | Women | | | |
| --- | --- | --- | --- | --- | --- | --- | --- | --- |
|  | Disability-free  life expectancy | | Perceived health life expectancy | | Disability-free  life expectancy | | Perceived health life expectancy | |
|  | Official^a^ | Estimated^b^ | Official^a^ | Estimated^b^ | Official^a^ | Estimated^b^ | Official^a^ | Estimated^b^ |
| 0 | 64.7 | 64.8 | 68.8 | 68.9 | 65.2 | 65.4 | 68.4 | 68.6 |
| 5 | 60.5 | 60.6 | 64.0 | 64.1 | 61.0 | 61.2 | 63.6 | 63.8 |
| 10 | 55.8 | 55.9 | 59.1 | 59.1 | 56.3 | 56.4 | 58.6 | 58.8 |
| 15 | 51.0 | 51.1 | 54.2 | 54.2 | 51.5 | 51.6 | 53.7 | 53.9 |
| 20 | 46.2 | 46.3 | 49.4 | 49.4 | 46.7 | 46.8 | 48.9 | 49.1 |
| 25 | 41.4 | 41.5 | 44.7 | 44.7 | 41.9 | 42.1 | 44.3 | 44.5 |
| 30 | 36.8 | 36.8 | 40.1 | 40.1 | 37.2 | 37.4 | 39.7 | 39.9 |
| 35 | 32.1 | 32.1 | 35.6 | 35.6 | 32.6 | 32.8 | 35.1 | 35.3 |
| 40 | 27.5 | 27.5 | 31.1 | 31.1 | 28.0 | 28.1 | 30.4 | 30.6 |
| 45 | 23.0 | 23.1 | 26.6 | 26.6 | 23.5 | 23.6 | 25.9 | 26.1 |
| 50 | 18.8 | 18.8 | 22.4 | 22.4 | 19.1 | 19.3 | 21.7 | 21.9 |
| 55 | 14.9 | 15.0 | 18.4 | 18.4 | 15.0 | 15.2 | 17.6 | 17.8 |
| 60 | 11.5 | 11.6 | 14.7 | 14.7 | 11.5 | 11.6 | 13.9 | 14.1 |
| 65 | 8.5 | 8.6 | 11.3 | 11.3 | 8.5 | 8.7 | 10.5 | 10.7 |
| 70 | 6.2 | 6.3 | 8.2 | 8.2 | 6.1 | 6.3 | 7.5 | 7.7 |
| 75 | 4.2 | 4.4 | 5.7 | 5.8 | 4.5 | 4.7 | 5.2 | 5.5 |
| 80 | 2.9 | 3.1 | 3.9 | 4.0 | 3.3 | 3.5 | 3.6 | 3.8 |
| 85+ | 2.0 | 2.0 | 2.4 | 2.5 | 2.6 | 2.8 | 2.4 | 2.7 |

All the healthy life expectancy was estimated by Sullivan method

^a^Official statistics on healthy life expectancy of Korean population in 2016 from the Korean Statistical Information Service (KOSIS)

^b^Estimated healthy life expectancy (2014–2018) of the general population from this study
