# Supplementary material for: Life expectancy and healthy life expectancy of Korean registered disabled by disability type in 2014–2018: Korea National Rehabilitation Center database
Source: BMC Public Health. 2023 Sep 8;23:1750. doi: 10.1186/s12889-023-16682-9 (PMC10485940; doi:10.1186/s12889-023-16682-9)
Supplement: Supplementary file 6 — Additional file 6. Abbreviated life expectancy by type of disability and sex in Korea (2014–2018). [file 12889_2023_16682_MOESM6_ESM.docx]

Additional file 6. Abbreviated life expectancy by type of disability and sex in Korea (2014–2018)

|  | Type of disability | | | | | | | | | | | | | | | |
| --- | --- | --- | --- | --- | --- | --- | --- | --- | --- | --- | --- | --- | --- | --- | --- | --- |
|  | Impairment in external bodily functions | | | | | | | Internal organs | | | | | | Developmental disabilities | | Mental disorder |
|  | Hearing disability | | Visual disability | Physical disability | Speech disability | Facial disfigurement | Disability of brain Lesion | Epilepsy | Kidney dysfunction | Hepatic dysfunction | Cardiac dysfunction | Intestinal Fistula/ Urinary Fistula | Respiratory dysfunction | Intellectual disorder | Autism spectrum disorder |  |
| Age (years) | | | | | | | | | | | | | | | | |
| Male | | | | | | | | | | | | | | | | |
| First age^a^ | | 77.5 | 71.6 | 67.4 | 62.2 | 68.2 | 47.0 | 50.7 | 49.1 | 38.3 | 36.2 | 28.1 | 23.3 | 67.2 | 50.5 | 52.7 |
| 5 | 74.2 | | 69 | 65.5 | 61.4 | 67.2 | 48.1 | 51.6 | 49.7 | 39.5 | 38.2 | 29.6 | 25.5 | 65.6 | 52.0 |  |
| 10 | 69.5 | | 64.6 | 61.3 | 57.5 | 63 | 45.1 | 48.2 | 46.5 | 37.5 | 36 | 28.5 | 24.4 | 61.2 | 47.2 |  |
| 15 | 64.8 | | 60 | 57.1 | 53.4 | 58.8 | 41.8 | 44.7 | 43 | 35.2 | 33.5 | 27 | 23 | 56.7 | 42.3 | 50.5^b^ |
| 20 | 60.1 | | 55.6 | 52.9 | 49.4 | 54.6 | 38.8 | 41.3 | 39.7 | 33.1 | 31.2 | 25.7 | 21.9 | 52.3 | 37.5 | 46.6 |
| 25 | 55.5 | | 51.2 | 48.8 | 45.5 | 50.4 | 35.7 | 37.9 | 36.4 | 31 | 29 | 24.4 | 20.7 | 48 | 32.7 | 42.7 |
| 30 | 50.8 | | 46.7 | 44.6 | 41.5 | 46.2 | 32.5 | 34.5 | 33 | 28.7 | 26.5 | 22.9 | 19.3 | 43.6 | 28.0 | 38.8 |
| 35 | 46.1 | | 42.2 | 40.4 | 37.4 | 42.0 | 29.2 | 30.9 | 29.5 | 26.2 | 23.9 | 21.2 | 17.7 | 39.1 | 23.2 | 34.8 |
| 40 | 41.5 | | 37.7 | 36.2 | 33.3 | 37.8 | 25.8 | 27.3 | 26 | 23.7 | 21.2 | 19.3 | 15.9 | 34.7 | 18.4 | 30.8 |
| 45 | 36.9 | | 33.4 | 32.1 | 29.4 | 33.7 | 22.7 | 23.9 | 22.7 | 21.3 | 18.7 | 17.6 | 14.3 | 30.4 | 14.0 | 27 |
| 50 | 32.5 | | 29.2 | 28.2 | 25.7 | 29.8 | 19.8 | 20.8 | 19.7 | 19.1 | 16.4 | 16.1 | 12.9 | 26.4 | 10.0 | 23.5 |
| 55 | 28.2 | | 25.3 | 24.6 | 22.3 | 26.1 | 17.2 | 18 | 16.9 | 17.1 | 14.4 | 14.7 | 11.6 | 22.5 | 7.0 | 20.2 |
| 60 | 24.1 | | 21.5 | 21.1 | 19.0 | 22.5 | 14.8 | 15.3 | 14.3 | 15.1 | 12.5 | 13.3 | 10.4 | 18.9 | 4.7^c^ | 17.1 |
| 65 | 20.2 | | 17.9 | 17.8 | 16.0 | 19.1 | 12.5 | 12.8 | 11.9 | 13.3 | 10.7 | 11.9 | 9.2 | 15.5 |  | 14.2 |
| 70 | 16.4 | | 14.5 | 14.6 | 13.0 | 15.9 | 10.3 | 10.5 | 9.7 | 11.5 | 9.0 | 10.6 | 8.1 | 12.3 |  | 11.4 |
| 75 | 13.1 | | 11.5 | 11.9 | 10.5 | 13 | 8.5 | 8.5 | 7.8 | 9.9 | 7.6 | 9.4 | 7.1 | 9.6 |  | 9.1 |
| 80 | 10.3 | | 9.1 | 9.6 | 8.5 | 10.6 | 7.1 | 7 | 6.3 | 8.7 | 6.4 | 8.4 | 6.3 | 7.4 |  | 7.3 |
| 85+ | 8.0 | | 7.1 | 7.7 | 6.9 | 8.6 | 5.9 | 5.8 | 5.3 | 7.6 | 5.6 | 7.5 | 5.6 | 5.8 |  | 5.9 |
| Female | | | | | | | | | | | | | | | | |
| First age^a^ | 83.1 | | 75.6 | 74.4 | 66.8 | 62.0 | 48.9 | 58.4 | 46.4 | 45.4 | 35.9 | 23.6 | 18.7 | 71.3 | 19.7 | 58.1 |
| 5 | 80.5 | | 74.3 | 73.2 | 66.4 | 61.8 | 50.1 | 59.4 | 50.3 | 46.6 | 38.4 | 24.9 | 20 | 70.4 | 47.0 |  |
| 10 | 76.0 | | 70.1 | 69.2 | 62.7 | 58.6 | 47.9 | 56.1 | 47.7 | 44.8 | 37.0 | 24.6 | 19.9 | 66.3 | 42.8 |  |
| 15 | 71.3 | | 65.7 | 64.9 | 58.7 | 55 | 45.2 | 52.4 | 44.6 | 42.4 | 35.0 | 23.9 | 19.5 | 61.9 | 38.1 | 56.1^a^ |
| 20 | 66.6 | | 61.1 | 60.4 | 54.5 | 51.2 | 42.1 | 48.4 | 41.2 | 39.8 | 32.5 | 22.9 | 18.7 | 57.4 | 33.2 | 52.1 |
| 25 | 62.0 | | 56.8 | 56.1 | 50.4 | 47.6 | 39.3 | 44.7 | 38.1 | 37.4 | 30.4 | 22.2 | 18.1 | 53.1 | 28.7 | 48.3 |
| 30 | 57.3 | | 52.3 | 51.8 | 46.3 | 43.9 | 36.4 | 40.9 | 34.8 | 34.8 | 28.0 | 21.2 | 17.4 | 48.7 | 24.0 | 44.3 |
| 35 | 52.7 | | 47.9 | 47.5 | 42.2 | 40.2 | 33.5 | 37.1 | 31.6 | 32.3 | 25.7 | 20.2 | 16.6 | 44.3 | 19.5 | 40.4 |
| 40 | 48.1 | | 43.6 | 43.2 | 38.2 | 36.6 | 30.6 | 33.4 | 28.4 | 29.7 | 23.5 | 19.2 | 15.8 | 40.0 | 15.3 | 36.6 |
| 45 | 43.5 | | 39.3 | 39 | 34.3 | 33 | 27.8 | 29.9 | 25.4 | 27.3 | 21.3 | 18.2 | 15.1 | 35.8 | 11.6 | 32.9 |
| 50 | 39 | | 35 | 34.9 | 30.5 | 29.6 | 25.1 | 26.4 | 22.5 | 24.8 | 19.2 | 17.2 | 14.3 | 31.7 | 8.6 | 29.3 |
| 55 | 34.6 | | 30.9 | 30.8 | 26.7 | 26.1 | 22.4 | 23 | 19.7 | 22.4 | 17.1 | 16.2 | 13.5 | 27.6 | 6.3 | 25.7 |
| 60 | 30.1 | | 26.7 | 26.7 | 22.9 | 22.7 | 19.5 | 19.5 | 16.7 | 19.8 | 14.9 | 15 | 12.5 | 23.5 | 4.3^b^ | 22 |
| 65 | 25.7 | | 22.6 | 22.7 | 19.2 | 19.2 | 16.8 | 16.2 | 13.9 | 17.3 | 12.7 | 13.7 | 11.5 | 19.5 |  | 18.5 |
| 70 | 21.4 | | 18.6 | 18.8 | 15.6 | 15.9 | 14 | 12.9 | 11.1 | 14.7 | 10.5 | 12.4 | 10.4 | 15.7 |  | 15 |
| 75 | 17.3 | | 14.9 | 15.1 | 12.3 | 12.9 | 11.5 | 10 | 8.7 | 12.4 | 8.6 | 11.1 | 9.4 | 12.1 |  | 11.9 |
| 80 | 13.6 | | 11.5 | 11.9 | 9.4 | 10.2 | 9.3 | 7.6 | 6.7 | 10.3 | 6.9 | 9.9 | 8.4 | 9.1 |  | 9.2 |
| 85 | 10.4 | | 8.8 | 9.1 | 7.1 | 8.0 | 7.5 | 5.7 | 5.1 | 8.6 | 5.6 | 8.8 | 7.5 | 6.7 |  | 7.0 |

^a^Age at the disability registration by the law in Korea for people with disabilities (They are shown in Table 1)

^b^Aged from 13 to 15 year

^c^Aged ≥60 years
