# Supplementary material for: Life expectancy and healthy life expectancy of Korean registered disabled by disability type in 2014–2018: Korea National Rehabilitation Center database
Source: BMC Public Health. 2023 Sep 8;23:1750. doi: 10.1186/s12889-023-16682-9 (PMC10485940; doi:10.1186/s12889-023-16682-9)
Supplement: Supplementary file 7 — Additional file 7. Abbreviated healthy life expectancy using the National Health Insurance System database by type of disability and sex in Korea (2014–2018). [file 12889_2023_16682_MOESM7_ESM.docx]

Additional file 7. Abbreviated healthy life expectancy using the National Health Insurance System database by type of disability and sex in Korea (2014–2018)

|  | Type of disability | | | | | | | | | | | | | | | | |
| --- | --- | --- | --- | --- | --- | --- | --- | --- | --- | --- | --- | --- | --- | --- | --- | --- | --- |
|  | Impairment in external bodily functions | | | | | | Internal organs | | | | | | | Developmental disabilities | | | Mental disorder |
|  | Hearing disability | Visual disability | Physical disability | Speech disability | Facial disfigurement | Disability of brain Lesion | Epilepsy | Kidney dysfunction | Hepatic dysfunction | Cardiac dysfunction | Intestinal Fistula/ Urinary Fistula | Respiratory dysfunction | Intellectual disorder | | Autism spectrum disorder | |  |
| Age (years) | | | | | | | | | | | | | | | | | |
| Male | | | | | | | | | | | | | | | | | |
| First age^a^ | 68.6 | 64.7 | 57.5 | 53.8 | 55.3 | 35.0 | 41.0 | 34.7 | 29.9 | 28.8 | 17.7 | 15.1 | 56.8 | | 47.2 | 32.2 | |
| 5 | 66.3 | 62.6 | 57.0 | 53.2 | 55.2 | 36.9 | 42.6 | 36.1 | 31.7 | 31.9 | 21.4 | 17.4 | 55.2 | | 48.5 |  | |
| 10 | 61.8 | 58.4 | 53.4 | 49.4 | 52.5 | 34.8 | 40.3 | 34.2 | 29.9 | 30.4 | 20.8 | 17.7 | 51.0 | | 43.8 |  | |
| 15 | 57.2 | 54.0 | 49.6 | 45.4 | 50.8 | 32.0 | 37.5 | 32.1 | 27.2 | 28.2 | 19.4 | 17.1 | 46.6 | | 38.9 | 30.9^b^ | |
| 20 | 52.7 | 49.7 | 46.0 | 41.4 | 47.0 | 29.0 | 34.5 | 29.5 | 25.0 | 25.9 | 19.0 | 15.2 | 42.4 | | 34.3 | 28.2 | |
| 25 | 48.2 | 45.4 | 42.2 | 37.8 | 43.1 | 26.1 | 31.3 | 26.9 | 22.7 | 23.6 | 18.5 | 15.6 | 38.2 | | 29.7 | 25.5 | |
| 30 | 43.6 | 41.0 | 38.2 | 33.8 | 39.5 | 23.1 | 28.2 | 24.3 | 20.6 | 21.2 | 16.9 | 14.7 | 34.1 | | 25.1 | 22.8 | |
| 35 | 39.0 | 36.6 | 34.2 | 29.8 | 35.5 | 20.2 | 24.9 | 21.4 | 18.8 | 18.8 | 16.4 | 12.9 | 30.1 | | 20.6 | 19.9 | |
| 40 | 34.5 | 32.3 | 30.2 | 26.0 | 31.4 | 17.3 | 21.6 | 18.5 | 17.4 | 16.4 | 14.9 | 11.4 | 26.2 | | 16.1 | 17.1 | |
| 45 | 30.1 | 28.1 | 26.3 | 22.3 | 27.4 | 14.9 | 18.7 | 15.8 | 15.9 | 14.1 | 13.4 | 9.9 | 22.4 | | 2.6^c^ | 14.4 | |
| 50 | 25.9 | 24.1 | 22.6 | 19.1 | 23.7 | 12.9 | 15.8 | 13.2 | 14.4 | 12.4 | 12.2 | 9.1 | 19.0 | |  | 12.1 | |
| 55 | 21.9 | 20.4 | 19.1 | 16.2 | 20.1 | 11.2 | 13.3 | 11.0 | 12.8 | 11.0 | 11.1 | 8.1 | 15.8 | |  | 10.1 | |
| 60 | 18.0 | 16.8 | 15.8 | 13.5 | 16.7 | 9.6 | 11.1 | 9.0 | 11.1 | 9.5 | 9.9 | 7.0 | 12.9 | |  | 8.3 | |
| 65 | 14.3 | 13.4 | 12.6 | 11.0 | 13.6 | 8.0 | 9.0 | 7.1 | 9.5 | 7.8 | 8.7 | 6.0 | 10.3 | |  | 6.8 | |
| 70 | 10.8 | 10.1 | 9.6 | 8.6 | 10.3 | 6.3 | 6.9 | 5.4 | 7.5 | 6.3 | 7.3 | 4.9 | 7.8 | |  | 5.5 | |
| 75 | 7.5 | 7.1 | 6.8 | 6.2 | 7.2 | 4.7 | 5.4 | 3.9 | 5.3 | 4.7 | 5.8 | 4.0 | 5.6 | |  | 4.1 | |
| 80 | 4.3 | 4.1 | 4.0 | 3.8 | 3.8 | 3.0 | 3.3 | 2.5 | 2.8 | 3.1 | 3.8 | 2.8 | 3.6 | |  | 2.9 | |
| 85+ | 0.8 | 0.8 | 0.8 | 0.7 | 0.0 | 0.7 | 1.1 | 0.5 | 0.0 | 0.6 | 0.8 | 0.7 | 0.8 | |  | 0.8 | |
| Female |  |  |  |  |  |  |  |  |  |  |  |  |  | |  |  | |
| First age^a^ | 70.1 | 65.2 | 59.5 | 56.6 | 47.8 | 35.7 | 46.3 | 30.9 | 34.7 | 27.0 | 11.1 | 10.8 | 59.7 | | 19.1 | 34.7 | |
| 5 | 68.3 | 68.1 | 60.0 | 56.4 | 48.9 | 37.5 | 47.4 | 35.3 | 36.5 | 31.6 | 13.5 | 13.4 | 58.8 | | 45.7 |  | |
| 10 | 64.0 | 64.2 | 56.7 | 52.9 | 47.8 | 35.9 | 45.2 | 34.1 | 34.6 | 30.6 | 15.7 | 15.3 | 54.8 | | 41.6 |  | |
| 15 | 59.5 | 59.9 | 53.1 | 48.9 | 47.1 | 33.5 | 41.6 | 31.7 | 32.4 | 28.4 | 16.8 | 14.9 | 50.6 | | 37.1 | 34.6 | |
| 20 | 54.8 | 55.5 | 49.1 | 44.8 | 43.6 | 30.4 | 38.1 | 29.6 | 29.8 | 26.1 | 15.8 | 15.1 | 46.3 | | 32.3 | 32.3 | |
| 25 | 50.3 | 51.3 | 45.0 | 41.1 | 40.5 | 27.5 | 34.6 | 27.2 | 27.3 | 23.9 | 16.2 | 14.1 | 42.1 | | 27.9 | 29.5 | |
| 30 | 45.7 | 47.0 | 40.9 | 37.1 | 36.9 | 24.7 | 31.4 | 24.7 | 25.0 | 21.9 | 15.2 | 13.0 | 38.0 | | 23.5 | 27.0 | |
| 35 | 41.3 | 42.8 | 36.9 | 33.3 | 34.0 | 22.0 | 27.9 | 22.2 | 22.7 | 19.9 | 14.6 | 11.8 | 34.0 | | 19.2 | 24.2 | |
| 40 | 36.8 | 38.6 | 32.9 | 29.5 | 30.4 | 19.4 | 24.6 | 19.6 | 21.1 | 17.7 | 13.9 | 11.3 | 30.0 | | 15.1 | 21.5 | |
| 45 | 32.4 | 34.6 | 28.9 | 25.7 | 27.0 | 17.1 | 21.3 | 17.1 | 19.6 | 15.9 | 13.5 | 10.6 | 26.2 | | 11.6 | 18.8 | |
| 50 | 28.0 | 30.6 | 25.0 | 22.2 | 23.7 | 14.9 | 18.0 | 14.6 | 17.9 | 14.0 | 12.9 | 9.7 | 22.5 | | 8.6 | 16.1 | |
| 55 | 23.8 | 26.8 | 21.3 | 18.8 | 20.5 | 13.0 | 15.0 | 12.2 | 15.8 | 11.9 | 12.0 | 8.8 | 19.0 | | 6.3 | 13.6 | |
| 60 | 19.7 | 23.0 | 17.6 | 15.4 | 17.3 | 11.0 | 12.0 | 9.8 | 13.5 | 10.2 | 10.9 | 7.9 | 15.5 | | 4.3 | 11.3 | |
| 65 | 15.6 | 19.4 | 14.0 | 12.1 | 14.2 | 8.9 | 9.2 | 7.6 | 11.1 | 8.4 | 9.5 | 6.8 | 12.2 | | 3.1 | 9.1 | |
| 70 | 11.7 | 15.9 | 10.5 | 9.1 | 11.0 | 6.8 | 6.3 | 5.5 | 8.7 | 6.6 | 7.9 | 5.4 | 9.2 | | 2.8 | 7.0 | |
| 75 | 7.9 | 12.8 | 7.1 | 6.2 | 7.7 | 4.7 | 4.0 | 3.6 | 5.9 | 4.7 | 5.9 | 4.3 | 6.3 | | 2.9 | 5.0 | |
| 80 | 4.3 | 10.2 | 4.0 | 3.5 | 4.4 | 2.8 | 2.1 | 2.1 | 3.2 | 3.1 | 3.7 | 2.7 | 3.6 | | 3.0 | 3.0 | |
| 85 | 0.7 | 8.5 | 0.7 | 0.6 | 1.0 | 0.6 | 0.0 | 0.5 | 0.0 | 0.7 | 0.8 | 0.5 | 0.7 | | 2.0 | 0.6 | |

^a^Age at the disability registration by the law in Korea for people with disabilities (They are shown in Table 1)

^b^Aged from 13 to 15 year

^c^Aged ≥60 years
